# Author Correction: Predicting Bordeaux red wine origins and vintages from raw gas chromatograms

**DOI:** 10.1038/s42004-024-01097-3

**Published:** 2024-01-11

**Authors:** Michael Schartner, Jeff M. Beck, Justine Laboyrie, Laurent Riquier, Stephanie Marchand, Alexandre Pouget

**Affiliations:** 1 Center for the Unknown, Champalimaud Institute, Lisbon, Portugal; 2 Duke University, Durham, NC USA; 3 Université de Bordeaux, ISVV, INRAE, UMR 1366 OENOLOGIE, 33140 Villenave d’Ornon, France; 4 Département des neurosciences fondamentales, Université de Genève, Genève, Suisse

**Keywords:** Analytical chemistry

Correction to: *Communications Chemistry* 10.1038/s42004-023-01051-9, published online 05 December 2023.

The original version of the Supplementary Material file published alongside this Article contained errors in Table S1.

Estate labels A, B, C, D, E, F and G were incorrectly assigned as V, A, S, F, T, G and B, respectively. As the wines are listed alphabetically in the Table according to their estate label, the order in which they were listed was also incorrect.

The correct version of the Supplementary Material file now accompanies the Article, and the original incorrect version of the Supplementary Material file is appended to this Correction.

### Supplementary information

Supplementary Material

