# Supplementary material for: Predicting Bordeaux red wine origins and vintages from raw gas chromatograms
Source: Commun Chem. 2023 Dec 5;6:247. doi: 10.1038/s42004-023-01051-9 (PMC10698164; doi:10.1038/s42004-023-01051-9)
Supplement: Supplementary file 2 — Supplementary Material [file 42004_2023_1051_MOESM2_ESM.pdf]

Supplementary Material

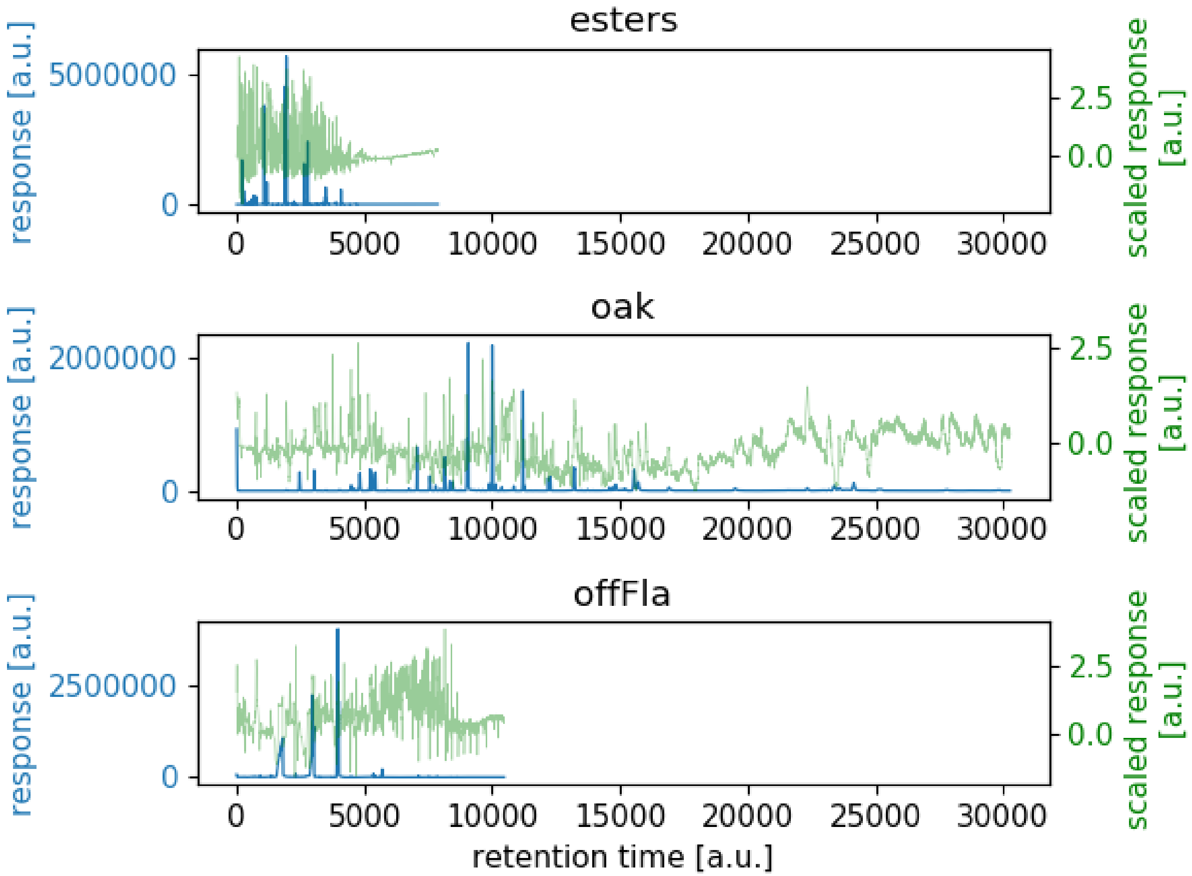

Figure S1: Example of chromatograms. We show, in blue, the three types of chromatograms for a single example wine. The green lines show the normalized spectrogram, $z(t) = (x(t) - u(t))/s(t)$ where $u(t)$ and $s(t)$ are the mean and standard deviation at retention time t across all wines and $x(t)$ is the non-normalized spectrogram. This scaling procedure reveals that seemingly flat portions of the chromatograms contain significant peaks, which might explain why the decoders assign large weights to these sections.

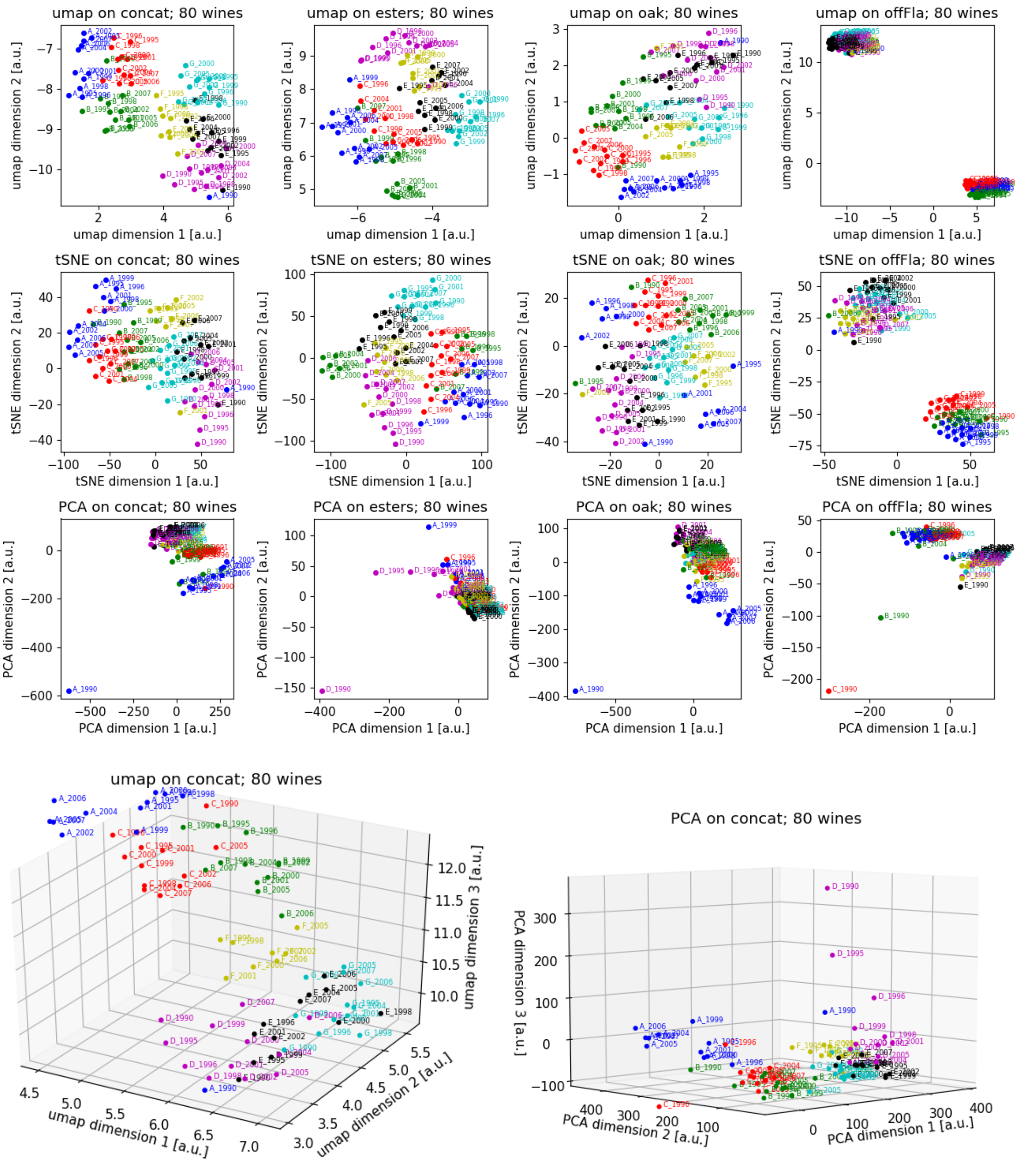

Figure S2: Additional dimensionality reduction plots. Dimensionality reduction of chromatograms using UMAP, t-SNE and PCA on all 3 chromatograms combined (concat), esters, oak and offFla only. Note that the results of UMAP and t-SNE on the concatenated chromatograms (top left panels) are not identical to the one shown in Fig. 1. This is because these algorithms yield slightly different results from run to run depending on the value of the initial seed. Critically, the main trends highlighted in the main text are still present: different

vintages from the same wine tend to cluster together, the right bank wines (A, C, B) stand on one side of the plot with the left bank ones on the other side, and the south-north axis of the Medoc region is still present (F-G: south, E-D: north). 3D plots: Dimensionality reduction of the concatenated chromatograms using UMAP (left) and PCA (right) in 3D did not reveal additional structure when compared to the 2D plots.

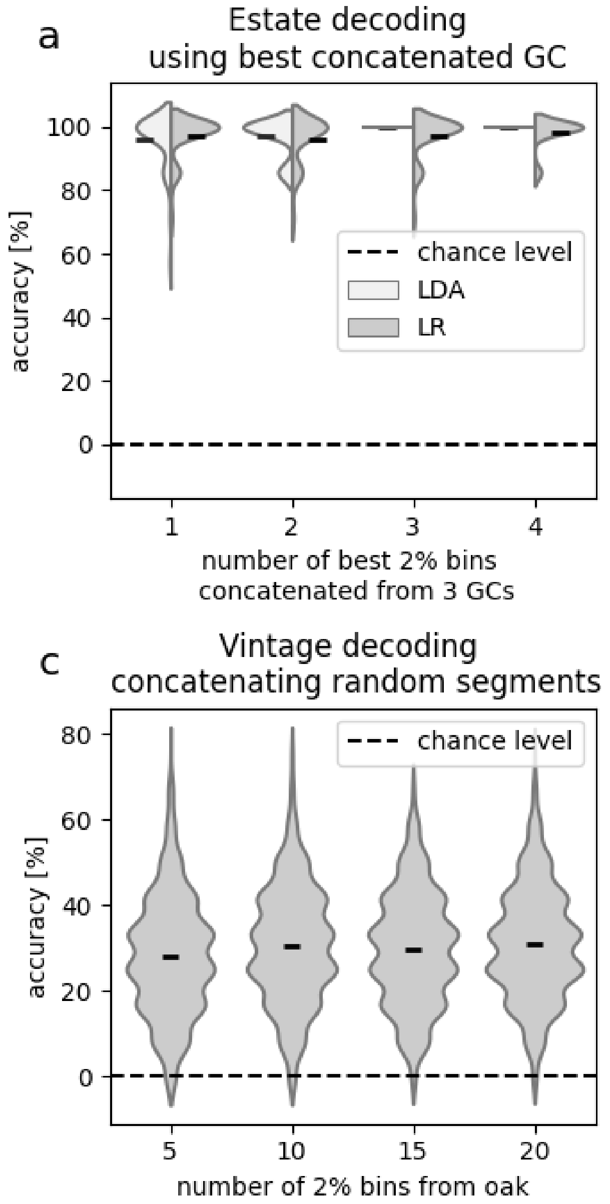

Figure S3: Estate and vintage decoding using the concatenation of the best segments from the survival algorithm. a) x-label being 2 means the shown accuracy is when concatenating the 2 “best” bins of data from each of the three chromatograms as input for the estate decoding. LDA has a perfect score when combining the 3 best such data bins from each of the three chromatograms. LR is unaffected, performing the same as for the full data. **b)** Same as **a** but for vintage decoding. **c)** Concatenating 5 randomly chosen 2% segments of oak as

input for the vintage decoder (LDA) led to a decoding accuracy of 0.3, i.e. slightly higher than the result obtained with the full oak chromatogram (0.24), showing that vintage-information is highly redundant in the chromatogram and a reduction of the data reduces overfitting. Note that the best vintage decoding performance, 0.5, was obtained by using the best 20% of oak data, found via the survival algorithm.

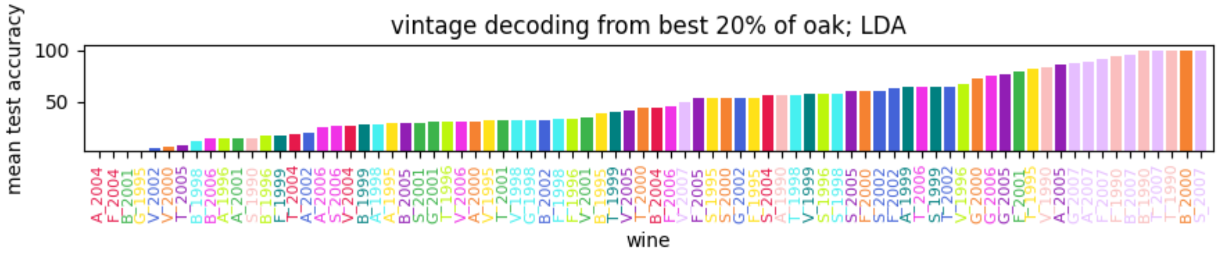

Figure S4: Vintage decodability per wine. The best 20% of the oak chromatogram - as found via the survival algorithm - was used to decode vintage. 100 times a test set - containing one wine for each vintage - was randomly chosen and classified using LDA, trained on the remaining wines. The mean test accuracy across these 100 splits is shown for each wine individually. The 2007 vintage was clearly easier to decode than other vintages (light purple color). The average accuracy across all wines is 0.5, which is double the accuracy that we found for LDA on the complete oak chromatogram.

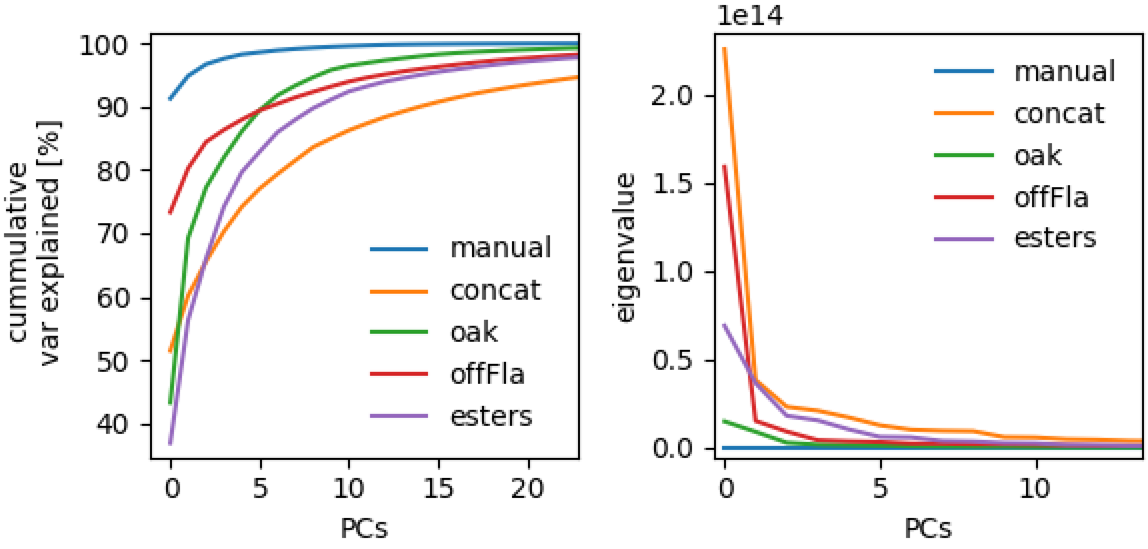

Figure S5: PCA of raw chromatograms and of spectra of manually extracted compounds concentrations (manual, blue line). 20 PCA components explain about 90% of the variance for the concatenated raw chromatograms. Less than 10 components are sufficient to explain 90% of the variance for oak, offFla and ester chromatograms. For the manually extracted concentrations (manual), the first component alone explains over 90% percent of the variance (95%). Right panel: scree plot showing the eigenvalue per principal component.

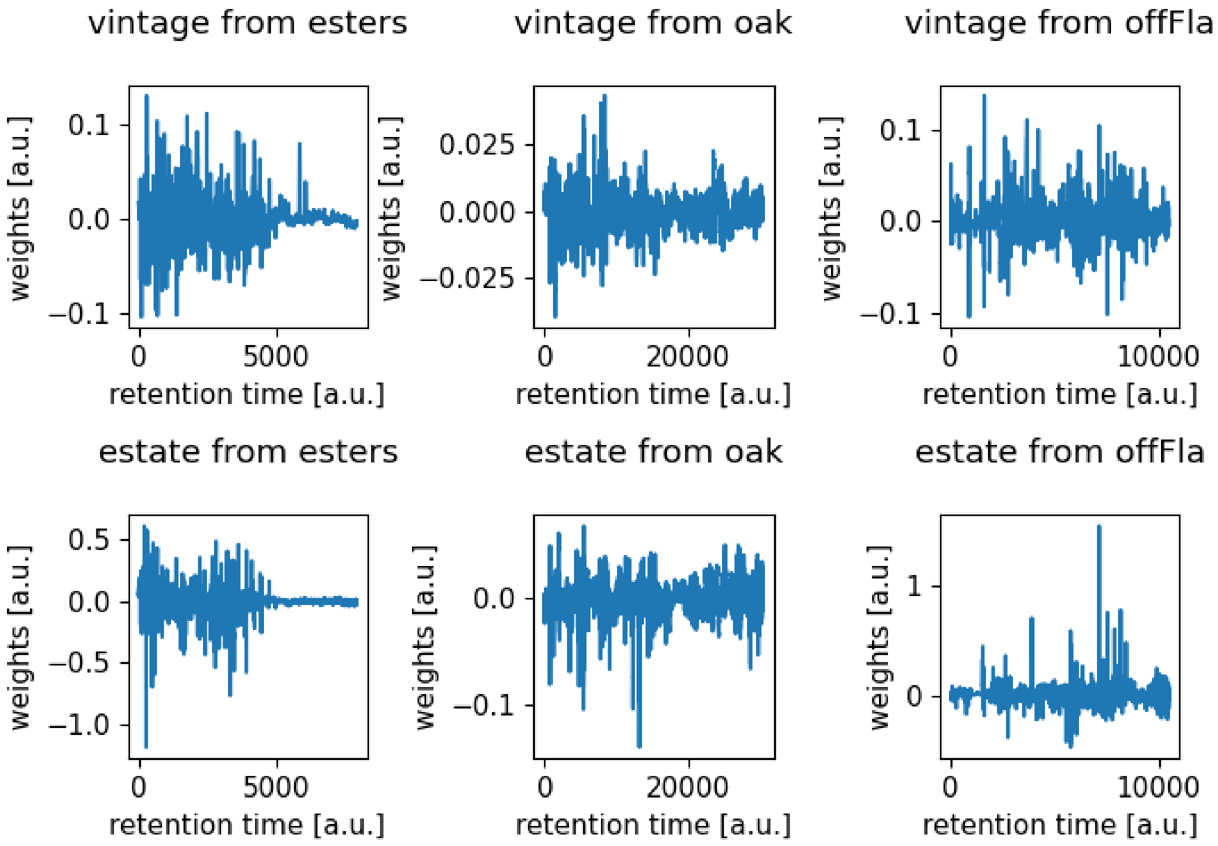

Figure S6: Example decoder weights. Decoding weight vectors for each variable (one example estate, one example vintage scores) and each chromatogram type (esters, oak and offFla). The weight vectors for one randomly chosen split of test/train are shown, being the LDA weights for one decoding class example (one estate or one vintage).

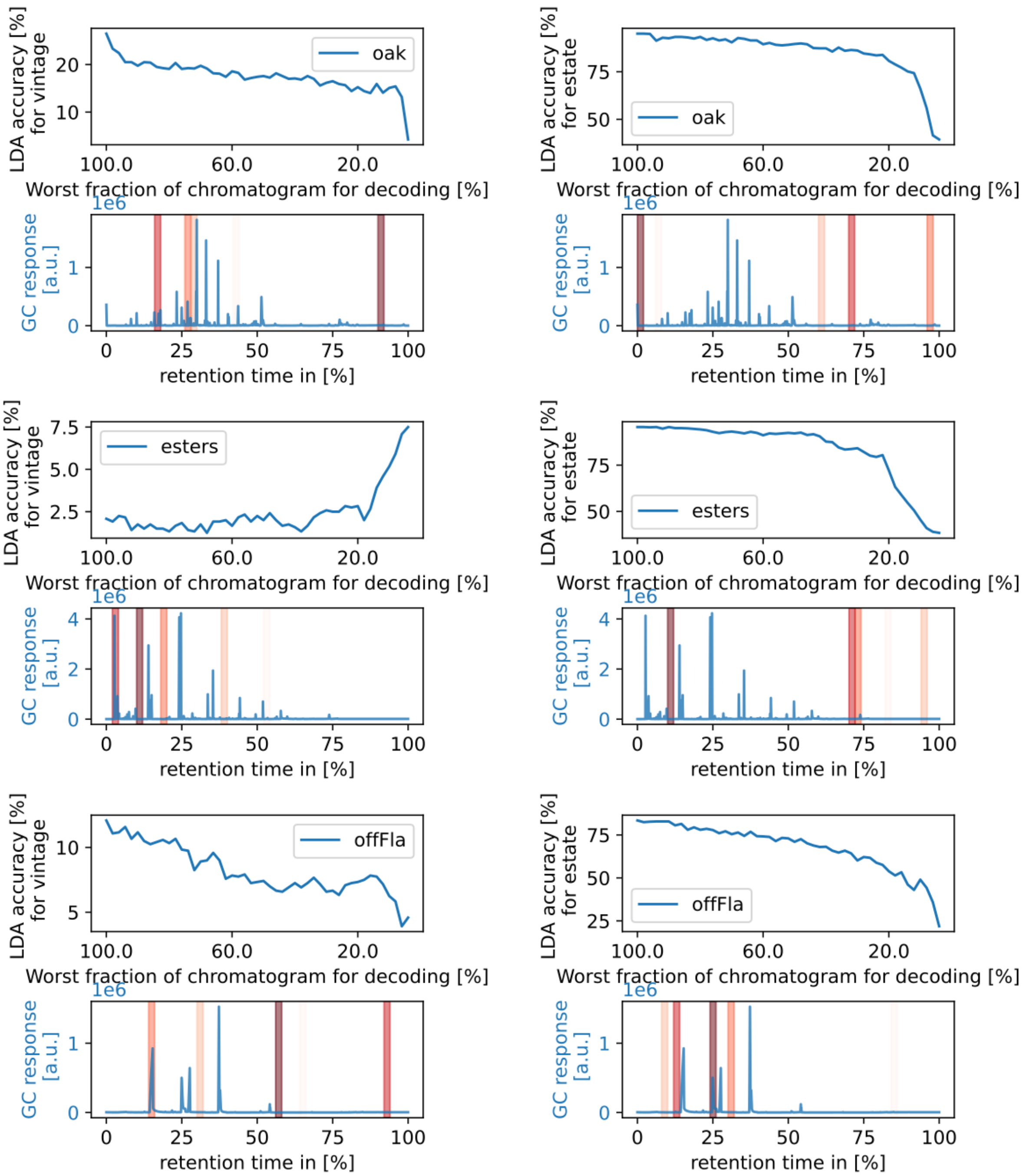

Figure S7: GC-specific "Survival" algorithm results, iteratively removing most informative bins. Same analysis as described in Fig. S3 but instead of removing the least informative bins iteratively, here the most informative bins were removed. Estate decoding from the three chromatograms still remains well above chance even for the worst 20% of the chromatogram, demonstrating that estate information is ubiquitously present across the chromatograms. The accuracy of decoding vintage from esters is increasing with the removal of worst bins, but note that vintage decoding for esters is below chance (0.08) throughout and thus only displays that there is no information about vintage in esters.

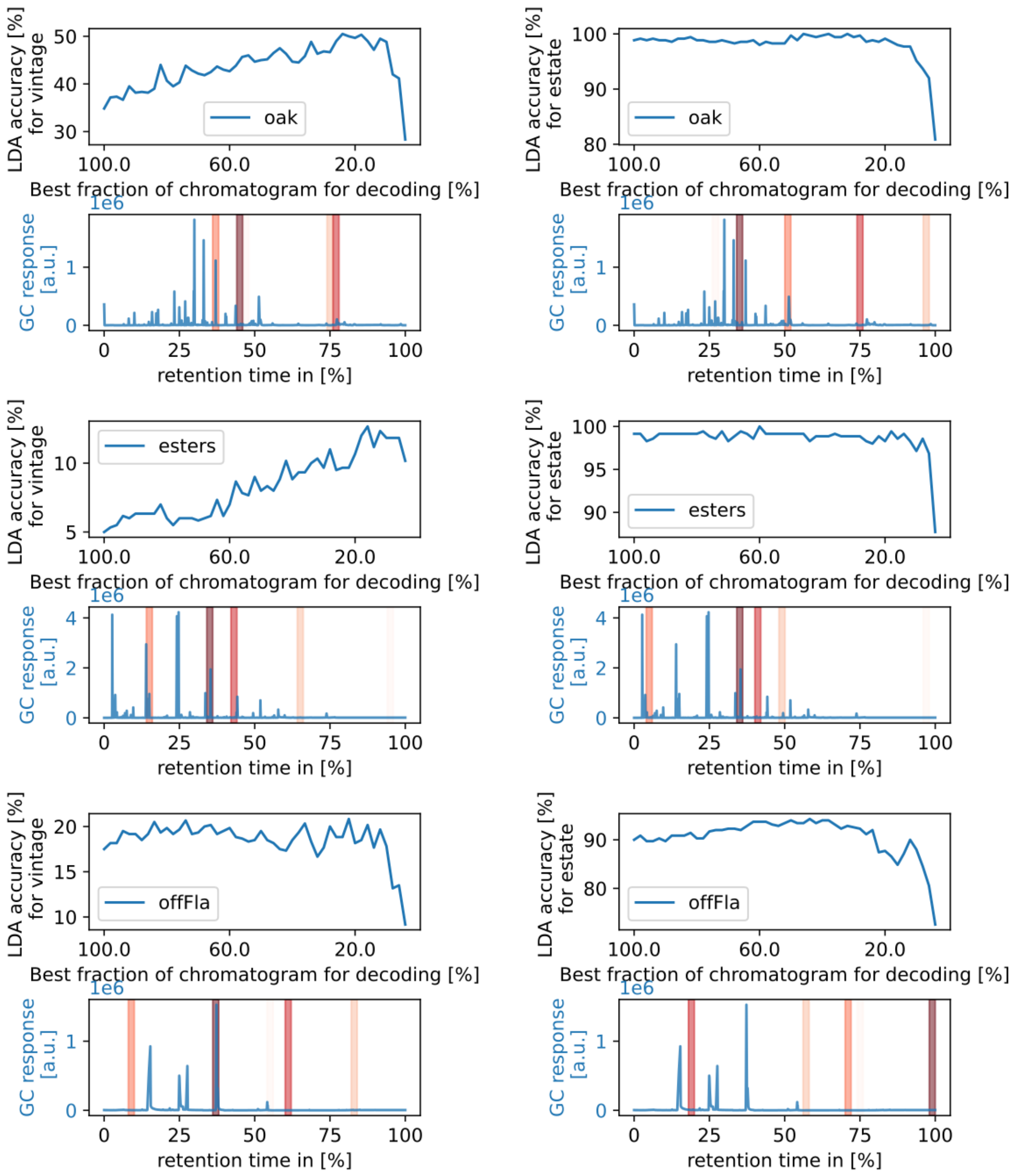

Figure S8: GC-specific “Survival” algorithm results, iteratively removing least informative bins. Each pair of plots shows, on top, the decoding accuracy (using LDA) as a function of the

best fraction of the chromatogram (analogous to Fig. 3a) and, at the bottom, the corresponding spectrogram with the best bins highlighted in red (darker for later removed). The left column of plots corresponds to vintage while the right column shows the results for estate identity. Bin size was chosen as 2% of the chromatogram (note it was 1% for esters shown in Fig. 3a). Importantly, in all cases, using a small fraction of the data (20% for oak, 10% for offFla) leads to decoding performance at least as good as for the whole chromatogram. In fact, in the case of vintage decoding from oak, the removal of uninformative sections of the chromatogram increased the decoding performance substantially.

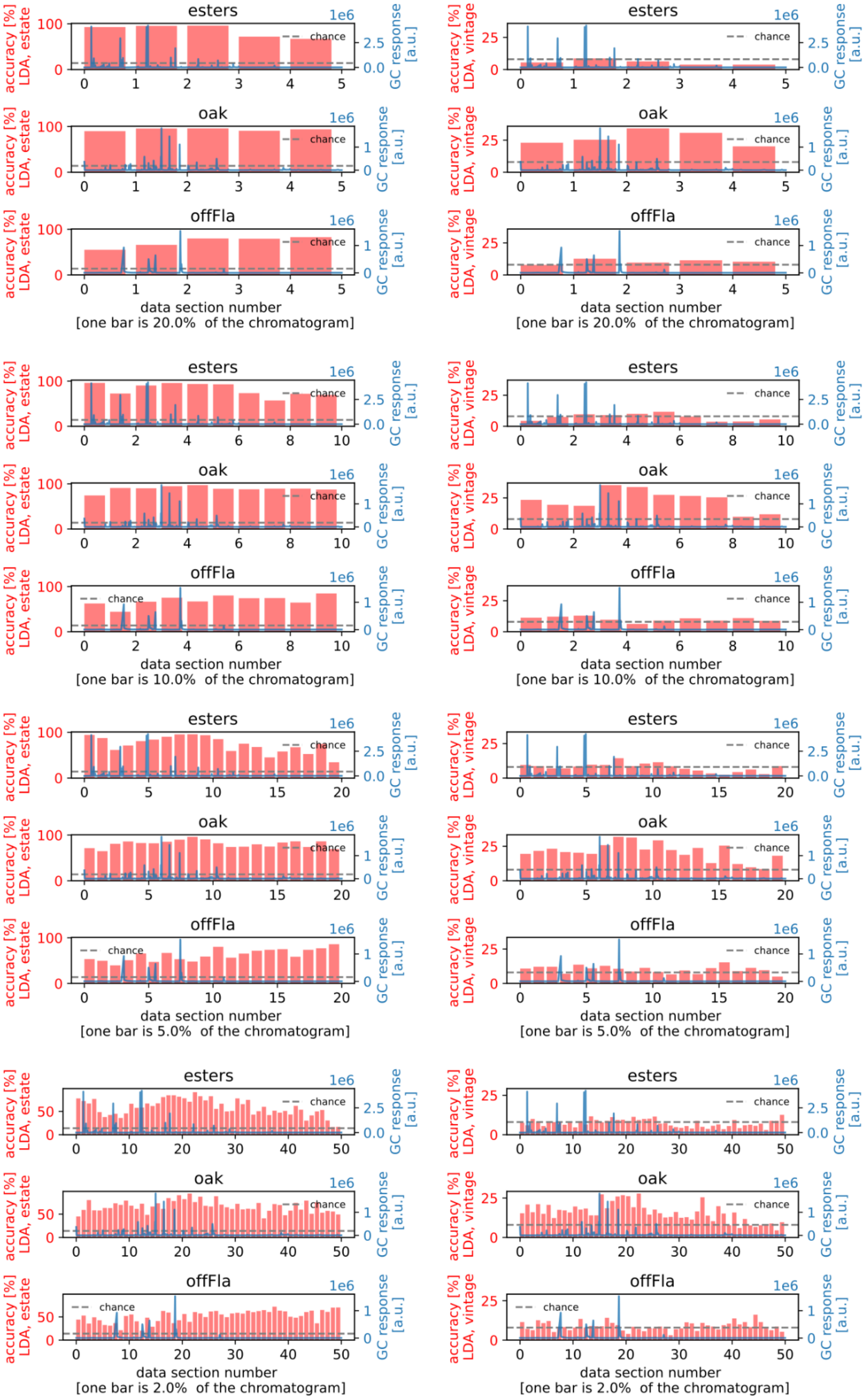
esters
oak
offFla
chance
1e6
accuracy [%] LDA, estate
accuracy [%] LDA, vintage
GC response [a.u.]
data section number
[one bar is 20.0% of the chromatogram]
[one bar is 10.0% of the chromatogram]
[one bar is 5.0% of the chromatogram]
[one bar is 2.0% of the chromatogram]

Figure S9: Estate and vintage decoding accuracy by section of the chromatogram. Analogous to Fig. 3 in the main text with different section sizes, dividing the chromatograms into 5, 10, 20 and 50 sections (see x-axis in above panels). Across different section sizes, the homogeneous decoding accuracy with section position remains, however some variance with section position is visible for all section sizes, for example, the first half of the ester chromatogram seems to hold more information about wine estate than the second half. Note also that the maximal decoding accuracy depends on the section size, with larger sections having larger decoding accuracy. Further, the oak chromatogram is 3 times as long as the esters or offFla (offFla) ones, explaining the overall higher decoding scores when using small data sections in percent.

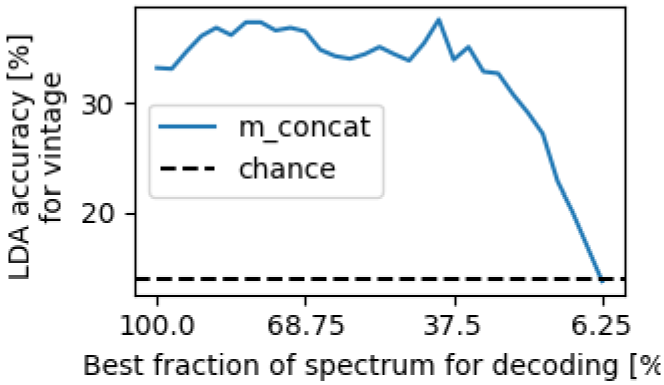

Figure S11: Vintage decoding "survival of the fittest" for concatenated compounds. When removing the least vintage-informative of the 32 compounds of m_concat at a time, decoding accuracy increases up to 37% when leaving the 12 best compounds, then sharply decreases.

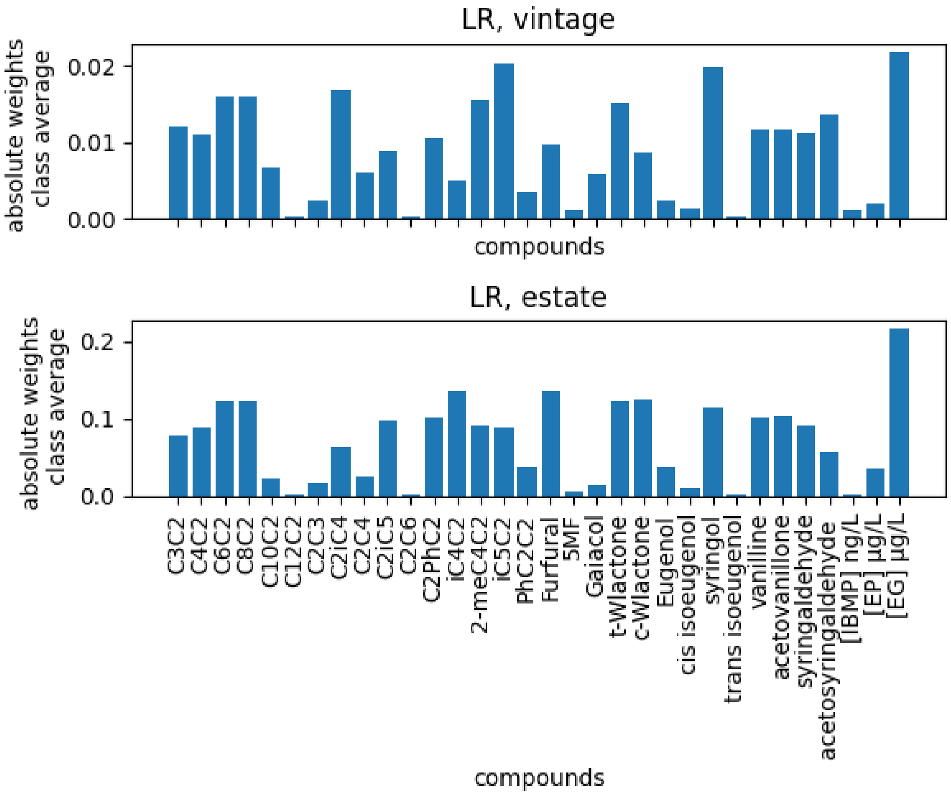

Figure S11: Regression weights are homogeneous across compounds. Training a logistic regression classifier (LR) on all wines, using the 32 compounds, results in similar weights for vintage (top) and estate (bottom) decoding, taking the absolute value of weights and averaging them across classes.

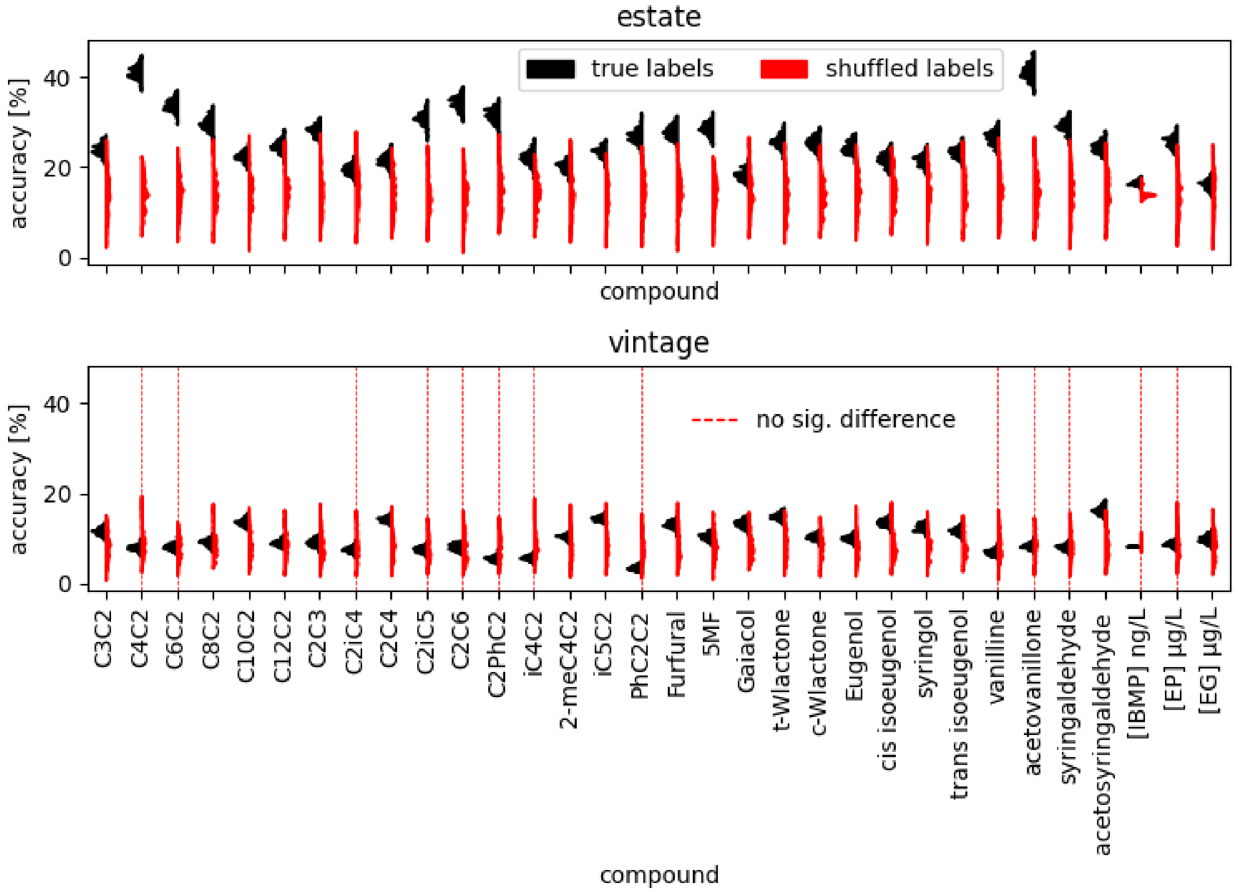

Figure S12: Estate and vintage decoding from individual chemical compounds. a) Estate decoding accuracy (using LR) for each of the 32 chemical compounds, scoring for all above chance, when comparing the distribution of scores obtained with the true estate labels to that obtained after shuffling labels 150 times (red on right for each compound). Distributions are significantly different if a one-sided t-test results in $p < 0.01$ (indicated by the absence of a red vertical line). **b)** vintage decoding is much weaker, insignificantly different from the null distribution for 13/32 compounds (note the maximum performance is only 18%).

	estate	vintage	CS [%]	M [%]	CF [%]	PV [%]
**0**	A	1995	0.000	0.470	0.530	0.000
**1**	A	1996	0.000	0.440	0.560	0.000
**2**	A	1998	0.000	0.560	0.440	0.000
**3**	A	1999	0.000	0.610	0.380	0.000
**4**	A	2000	0.000	0.530	0.470	0.000
**5**	A	2001	0.000	0.680	0.320	0.000
**6**	A	2002	0.000	0.530	0.470	0.000
**7**	A	2004	0.000	0.470	0.530	0.000
**8**	A	2005	0.000	0.510	0.490	0.000
**9**	A	2006	0.010	0.540	0.450	0.000
**10**	A	2007	0.000	0.470	0.530	0.000
**11**	B	1990	0.000	0.500	0.500	0.000
**12**	B	1995	0.000	0.500	0.500	0.000
**13**	B	1996	0.000	0.500	0.500	0.000
**14**	B	1998	0.000	0.500	0.500	0.000
**15**	B	1999	0.000	0.500	0.500	0.000
**16**	B	2000	0.000	0.500	0.500	0.000
**17**	B	2001	0.000	0.500	0.500	0.000
**18**	B	2002	0.000	0.500	0.500	0.000
**19**	B	2004	0.000	0.500	0.500	0.000
**20**	B	2005	0.000	0.500	0.500	0.000
**21**	B	2006	0.000	0.500	0.500	0.000
**22**	B	2007	0.000	0.500	0.500	0.000
**23**	C	1990	0.000	1.000	0.000	0.000
**24**	C	1995	0.000	1.000	0.000	0.000
**25**	C	1996	0.000	1.000	0.000	0.000
**26**	C	1998	0.000	1.000	0.000	0.000
**27**	C	1999	0.000	1.000	0.000	0.000
**28**	C	2000	0.000	1.000	0.000	0.000
**29**	C	2001	0.000	1.000	0.000	0.000
**30**	C	2002	0.000	1.000	0.000	0.000
**31**	C	2004	0.000	1.000	0.000	0.000
**32**	C	2005	0.000	1.000	0.000	0.000
**33**	C	2006	0.000	1.000	0.000	0.000
**34**	C	2007	0.000	1.000	0.000	0.000
**35**	D	1990	0.640	0.360	0.000	0.000
**36**	D	1995	0.680	0.250	0.070	0.000
**37**	D	1996	0.840	0.080	0.080	0.000
**38**	D	1998	0.810	0.190	0.000	0.000
**39**	D	1999	0.740	0.185	0.060	0.015
**40**	D	2000	0.930	0.070	0.000	0.000
**41**	D	2001	0.865	0.135	0.000	0.000
**42**	D	2002	0.870	0.095	0.035	0.000
**43**	D	2004	0.905	0.090	0.000	0.005
**44**	D	2005	0.888	0.107	0.000	0.005
**45**	D	2006	0.820	0.160	0.000	0.020
**46**	D	2007	0.840	0.150	0.000	0.010
**47**	E	1990	0.780	0.170	0.040	0.010
**48**	E	1995	0.740	0.220	0.030	0.010
**49**	E	1996	0.750	0.210	0.030	0.010
**50**	E	1998	0.720	0.230	0.040	0.010
**51**	E	1999	0.700	0.250	0.040	0.010
**52**	E	2000	0.770	0.160	0.040	0.030
**53**	E	2001	0.791	0.183	0.011	0.015
**54**	E	2002	0.764	0.223	0.010	0.003
**55**	E	2004	0.884	0.114	0.000	0.002
**56**	E	2005	0.857	0.137	0.000	0.006
**57**	E	2006	0.915	0.075	0.005	0.005
**58**	E	2007	0.911	0.089	0.000	0.000
**59**	F	1995	0.750	0.170	0.030	0.050
**60**	F	1998	0.750	0.180	0.020	0.050
**61**	F	2000	0.790	0.100	0.040	0.070
**62**	F	2001	0.821	0.072	0.034	0.073
**63**	F	2002	0.860	0.080	0.020	0.040
**64**	F	2005	0.850	0.080	0.010	0.060
**65**	F	2006	0.900	0.040	0.020	0.040
**66**	F	2007	0.870	0.110	0.020	0.000
**67**	G	1990	0.310	0.545	0.145	0.000
**68**	G	1995	0.428	0.477	0.095	0.000
**69**	G	1996	0.388	0.497	0.115	0.000
**70**	G	1998	0.290	0.590	0.120	0.000
**71**	G	1999	0.267	0.617	0.116	0.000
**72**	G	2000	0.436	0.548	0.016	0.000
**73**	G	2001	0.366	0.521	0.113	0.000
**74**	G	2002	0.399	0.513	0.088	0.000
**75**	G	2004	0.188	0.608	0.189	0.015
**76**	G	2005	0.387	0.592	0.021	0.000
**77**	G	2006	0.406	0.574	0.020	0.000
**78**	G	2007	0.437	0.428	0.135	0.000

Table S1: For each wine the estate code, vintage and grape varietal composition is shown. Cabernet-Sauvignon (CS), Merlot (M), Cabernet-Franc (CF) and Petit-Verdot (PV).
